# Evaluation of Sodium Hyaluronate Lubricating Drops Used before Insertion of Contact Lenses on Symptomatology, Severity, and Intensity of Ocular Dryness

**DOI:** 10.5402/2012/762784

**Published:** 2012-09-02

**Authors:** Langis Michaud, Benoît Frenette

**Affiliations:** École d'Optométrie, Université de Montréal, 3744 Jean-Brillant, Montréal, QC, Canada H3T 1P1

## Abstract

*Purpose*. This study aimed to evaluate outcomes from the use of a sodium hyaluronate (SH)-based comfort drop, instilled before the insertion of contact lenses, in a population of symptomatic contact lens wearers. *Methods*. This was a cross-over, open-label, multi-sites study. Subjects were fitted with silicone hydrogel lenses and followed for two months. Before insertion of the lenses, SH drops was instilled in the lens for half of the group. The other half did the same on the second month. Objectives and subjective outcomes were measured and compared before from baseline with the ones collected after usage of SH drops.

## 1. Introduction 

 As mentioned in the *Report of the National Eye Institute* [1], dry eye is a disorder of the tear film due to tear deficiency or excessive tear evaporation, which causes damage to the interpalpebral ocular surface, and is associated with symptoms of ocular discomfort. Since contact lenses disrupt the integrity of the tear film and thin out the lipid layer, thus increasing tear evaporation, they can potentially cause symptoms of eye dryness in wearers [2].

 Chalmers et al. [3] found that ocular dryness affects 20% of wearers of new-generation silicone hydrogel contact lenses, as well as 24% of wearers of older-generation, low-oxygen permeable (pHEMA) lenses. Brennan and Efron [4] found that 75% of low-oxygen permeable lens wearers report a feeling of discomfort at the end of the day related to dry eye. In general, it is believed that more than 50% of contact lens wearers are symptomatic of dry eye [5]. They may also present with clinical signs of dryness, such as damage to the surface of the eye, as well as instability and/or hyperosmolarity of the tear film; however, the symptoms do not always correlate with the objective signs [1]. In all of these cases, the subject develops discomfort in the absence of any ocular pathology that could trigger the dryness. All other factors being discarded, the contact lens wear must therefore be considered as the most likely etiology of the subject's symptoms. 

 Subjects with induced ocular dryness by contact lens wear usually tend to drop out from this modality when the benefits of the lenses are outweighed by the discomfort they feel, especially during the last hours of wear, day after day. This is the main reason why 20% of low-oxygen permeable lens wearers and 13% of silicone hydrogel lens [6] wearers stop wearing contact lenses every year. This discontinuation rate represents a continuous loss for the industry and for eyecare professionals, and the quest to find new methods to prevent induced dryness of the ocular surface is an ongoing concern. 

 Several options have been explored, with varying degrees of success. Manufacturers first tried to deal with the materials used for contact lenses. Among other things, comfort depends on a given material's rate of on-eye dehydration [7–11], wettability [12, 13], and the presence of surface deposits [8–10]. Silicone hydrogel lenses are currently considered to be the material of choice compared with hydrogels. Clinically, while many benefits [14, 15] are recognized, studies have shown that silicone hydrogel lenses have not altered end-of-day discomfort, as described above, at least not as expected [16], with the exception of senofilcon A, which was found to improve comfort in adverse and challenging environments [17]. 

 When materials proved not to be a solution to the problem of dry eye, researchers turned their attention to the lens design (spherical versus aspherical), the edge profile (sharp and thin versus rounded and thicker), the fit of the lens on the eye (flat or steep), and the replacement rate (2 weeks versus 1 month). On many of these aspects, no definite conclusions have been reached. 

 Care products were also considered as triggering ocular dryness symptoms because they are chemical products that are known to cause short- and medium-term toxic and allergic reactions, leading to patient discomfort. Preservatives have been identified as the main components of these products that can trigger these reactions among contact lens wearers [18–22]. It is also known that specific biocides found in contact lens solutions can be related to toxic and allergic reactions that produce more severe symptoms in wearers. Accordingly, solutions containing polyhexanides, specifically polyaminopropyl biguanide (PAPB) and polyhexamethylene biguanide (PHMB), are known to increase the potential for adverse reactions [22]. Polyhexanides are polycationic agents with a relatively high molecular weight, which, in theory, should limit their absorption by the contact lens. However, studies have shown that certain materials can easily absorb a large quantity of polycationic agents during the lens soaking phase (at night) [20]. Other factors that can explain this absorption are the number and the nature of buffers in the solution and the presence of sodium chloride which determines the ionic strength of the lens-solution interaction.

 On insertion, the lens balances out with the surrounding ocular environment, and the biocides absorbed by the lens matrix are released into the eye, possibly contributing to a temporary alteration of the ocular surface, especially during the first four hours of wear [21]. Absorption and release rates vary depending on the agents and materials used, and not their surface treatment, which determines compatibility between certain solutions and certain materials [23]. For example, Aldox penetrates more than PHMB and is released more rapidly (between 0 and 60 min) into the eye after insertion of the contact lens [24]. 

 Any involvement of the ocular surface should theoretically be avoided. This has prompted certain authors to suggest that wearers neutralize the polycationic agents (such as PHMB) absorbed during soaking by adding a drop of anionic [25] solution to the lenses before insertion. The anionic-cationic reaction creates a chemically neutral environment, sparing the cornea from temporary structural changes during wear. This has been proven to contribute to increased patient comfort, even at the end of the day. The simple use of a lubricant in the morning would therefore significantly improve end-of-day symptoms by creating a chemically neutral environment in the cornea from the outset, thus eliminating the potential for toxic or allergic reactions. The only agent that has been tested in this regard and that has confirmed this hypothesis is carboxymethyl cellulose (CMC), an anionic polymer found in certain lubricants (Refresh, Allergan, Irivne, CA). CMC is a water-soluble synthetic cellulose derivative with marked hydrophilic and bioadhesive properties that promote faster repair of the eye surface [26]. 

 This study aims to evaluate the performance of sodium hyaluronate (SH) in the same way. In fact, it is believed that the use of this product, instilled in the lens, before insertion, would chemically neutralize the biocide effects on the ocular surface, helping to alleviate initial and long-term discomfort associated to contact lens wear. SH is a viscoelastic agent used in several eye care products, both topically and in surgery, to protect and maintain the integrity of ocular tissues [27]. 

## 2. Methods 

This was a two-month multicenter study. Sixty-four patients were recruited among 4 optometric offices in the greater Montreal area, each of them being selected as representative of an average optometric practice in North America, based on the number and type of patients seen. One optometrist per office was designated and appropriately trained to conduct the study, in order to maximize the interobserver agreement rate. Training and coordination of the study were the responsibility of l'École d'Optométrie de l'Université de Montréal. Regular contacts by email or phone were maintained during the study between the university coordinator (LM) and the associate researchers. This study received approval from the ethics committee for health sciences of Université de Montréal. 

 Inclusion and exclusion criteria of subjects are listed in Table 1. After being enrolled, subjects were asked to observe a wash-out period of 72 hours without contact lenses before the beginning of the study. Subjects had to be established wearers, for at least 3 months, of the silicone hydrogel lenses (comfilcon A-CooperVision, Fairport, NY). Subjects were then randomly assigned to four groups (A–D), based on the care regimen to be used for the length of the study A: Complete (AMO-Abbott, Irvine, CA); B, Opti-Free Replenish (Alcon, Forth Worth, TX); C: Renu Fresh (B&Lomb, Rochester, NY); D: Clear Care (Ciba Vision, Duluth, GA). 

 Once recruited, the subject was provided with a new pair of contact lens and the care regimen steps were reviewed, according to the manufacturer's recommendations. Lens fitting was revisited. There was only one base curve available for the lens, limiting the customization of the fit. However, every lens fitted had to be centered, to offer a movement of 0.2 mm maximum upon blinking, and did not expose the limbus in any manner, in any gaze. For half of the subjects, they were told to wear the lens for a month, on a daily wear basis, and to not use any drops prior to insertion of the lenses. For the other half, they were told to instill a sodium hyaluronate based comfort drop (Blink Contacts, Abbott-AMO, Irvine, CA) in the lens (not in the eye), before insertion. Nothing was said to subjects about potential clinical effects of this product not to justify its use before insertion. Usage of the comfort drops during the day was not authorized. At 1 month, subjects were seen back for a control exam and assessed.

 At the 1-month visit, a case history was performed to identify subjects' level of compliance with the protocol and to answer any questions that might be raised by them. Visual acuity was measured using an ETDRS high-contrast chart under dim standard illumination. A slit lamp exam was performed to evaluate conjunctival and corneal staining, conjunctival hyperemia, and tear break-up time (TBUT). Efron's grading scale was used to evaluate the presence and severity of clinical signs, with grades varying from 0 (absence) to 4 (severe). Fluorescein was observed using a cobalt blue filter and a yellow filter (Kodak Wratten Filter number 12) to reveal any contrasts. The same filters were also used to evaluate TBUT, which is measured 2 times and averaged. Patients were then asked to complete 2 different questionnaires.

 The first questionnaire used in this study is the *Contact Lens Dry Eye Questionnaire* (CLDEQ). It consists of 36 questions specific to symptoms of ocular dryness related to contact lens wear. There are nine symptom subscales: discomfort, dryness, visual changes, soreness and irritation, grittiness and scratchiness, foreign body sensation, burning, photophobia, and finally itching. For each of these subscales, any occurrence of the symptom was also evaluated in term of intensity at different times (2 hours after insertion, middle and end of the wearing period). This questionnaire had already been validated and is sensitive and specific at predicting a diagnosis of dry eye associated with the wearing of contact lenses [28]. In addition to the CLDEQ questionnaire, subjects were asked to complete a brief survey about their contact lens experience over the last month. This is made through a forced-choice questionnaire where the subject had to click a box to select the best answer. Questions of this questionnaire are related to the subjective evaluation of comfort, with the lenses on, at different times of the day (morning, afternoon, evening). Subjects have to select from 5 options, from very comfortable (score of 5) to very uncomfortable (score of 1).

 The 1-month visit ended with a review of the care regimen procedures with instructions to the subject to wear the lenses for at least 8 hours/day, 5 days/week, and not to sleep or take a nap while wearing contact lenses. 

 A new pair of lenses was then provided and subjects were asked to observe a 48 h00 wash-out period of time, without lenses worn. For the next month, the subjects that did not use SH were asked to put a drop of a sodium-hyaluronate- (SH-) based product (Blink contacts, AMO-Abbott, Irvine, Ca) on the lens before insertion. The other ones had to cease to use such a drop. Subjects were seen a month later for the final visit, and the same clinical procedures were repeated. The results provided with and without the use of SH drops were considered for comparison. 

 At the end of the second visit, subjects had to answer the CLDEQ and a comfort questionnaire again. The second questionnaire was slightly different from the first one, with an additional question about which care regimen they would prefer to use in the future (with or without SH drops). 

## 3. Results

Out of the 64 subjects recruited, 61 were kept for statistical analysis. One file was rejected due to missing clinical data at one visit; another one was discarded because the subject admitted to have worn his lenses on an extended-wear basis most of the month. A third subject failed to fill in the last CLDEQ questionnaire. 

### 3.1. Population Sample

 A total of 61 patients completed the study successfully (18 males (30%) and 43 females (70%)). The mean subject age was 25.0 (±7,3) years old (range 18–35). At the beginning of the study, subjects reported spending 5 hours/day, on average (3 hours at work and 2 hours outside) on computer work. Only 5/61 (8.1%) had received a positive diagnosis of dry eye in the past, while 19 of them (31.1%) self-reported suffering from ocular dryness periodically. Some others (37 subjects—60.6%) were uncertain on this issue, having had some symptoms in the past. Fifteen subjects were assigned to group A, thirteen to group B, seventeen to group C, and sixteen to group D. Based on the population sample, characteristics of each group were found to be similar. 

For the following, visit 1 (V1) is related to the clinical results obtained when the subject was free of SH drops while visit 2 (V2) represents the results obtained after its usage. 

### 3.2. Monthly Hours of Wear

On average, a total of 76 (±22.00) hours of wear/week was estimated at V1 which is slightly less than 81 (±29.50) hours/week for V2. However, this difference is not considered statistically significant (*t*-test, *P* = 0.12). 

### 3.3. Clinical Findings

Table 2 shows the results for corneal and conjunctival staining, conjunctival hyperemia, and tear break-up time (TBUT). Corneal staining median values were reduced from V1 to V2. There was a significant decrease in the presence and the severity of corneal staining. More subjects (41 versus 38) showed no staining with the use of drops. Also, 50% fewer subjects showed clinically significant staining (grades 2-3) with this usage. Results for the right eyes mirrored the findings for the left eyes. The differences on the presence of the corneal staining show a tendency for statistical significance (*F*_(2,122)_ = 0.6323; *P* = 0.05). No care regimen was associated with a significant increase in corneal staining. 

Conjunctival staining (grade 1) was noticed in both temporal and nasal quadrants for 24% of the subjects on visit 1 and 17% on visit 2. This ocular sign was markedly reduced with the use of SH drop before insertion of the lens. There was an increase of 50% of subjects (23 versus 14) showing no hyperemia at V2 compared to V1. Clinically significant hyperemia (grade 2 and over) was also markedly reduced (10 subjects at V1 versus 3 at V2). Moreover, a 2/3 reduction in the severity of the hyperemia is observed. This difference is statistically significant (Pearson, *F*_(2,122)_ = 3.1217; *P* = 0.04; *r* ^2^ = 0.0281). 

Overall, the average tear break-up time increased slightly at the second visit from 5.8 (±3.1) sec to 6.3 (±3.3) sec OD and from 5.8 (±3.2) sec to 6.5 (±3.5) sec OS. This difference is not considered statistically significant (OD *P* = 0,2372; OS *P* = 0,1598). On the other hand, when we paired results for the same individual (subject compared to himself from V1 to V2), there was an increase in the TBUT value that is considered statistically significant (Tukey-Kramer, *q* = 1,9801, *P* = 0,05 OD and *q* = 1,9765, *P* = 0,05 OS). Finally, visual acuity remained stable during the study (V1/V2: OD 1.1 (±0.2)/1.0 (±0.2); OS 1.1 (±0.1)/1.0 (±0.2)). 

### 3.4. CLDEQ Questionnaire

From this questionnaire, we kept for analysis each symptom that was reported by at least 10% of the subjects, at one visit, intensity of the symptoms was analyzed (subquestions). Accordingly, discomfort (Q4), dryness (Q5), visual changes (Q6), soreness and irritation (Q7), grittiness and scratchiness (Q8), foreign body sensation (Q9), burning (Q10), photophobia (Q11), and itching (Q12) were analyzed. Grittiness and scratchiness (Q8), foreign body sensation (Q9), burning (Q10), photophobia (Q11), and itching (Q12) were not reported sufficiently to be considered for further analysis. 

### 3.5. Discomfort (Q4)

Table 3 shows the results. On average, there was a 10% increase in subjects reporting comfortable or very comfortable wear at the second visit. This difference is considered significant (chi square, *F*_(4,61)_ = 5.16; *P* = 0.0353; *r* ^2^  = 0.118). This result applies regardless of the care regimen used (*F*_(6,61)_ = 4.675*; P* = 0.15). Looking at the intensity of this symptom (Table 4), a greater difference is found at the beginning of the day, after 2 hours of wear (*P* = 0.00). This difference decreases by midday (*P* = 0.01) and at the end of the day (*P* = 0.03) but remains statistically significant at every time point.

### 3.6. Eye Dryness (Q5)

Table 5 provides the results. The difference is highly significant (chi square, *F*_(4,61)_ = 20.2312, *P* < 0.00) with a strong correlation (*r* ^2^ = 0.3191), showing a positive effect from the use of the SH drop, reducing the presence or the intensity of this symptom. In fact, there is a decrease of 20% of subjects reporting this symptom as constant at visit 2 compared to visit 1. Again here, no difference was found among the care system groups. The severity of the symptoms was also analyzed (CLDEQ questions 5b, c, and d).  Table 6 shows the results. Overall, there is a marked difference in the reduction of the severity of eye dryness felt by the patient (Q5b-chi square *F*_4,61_:  9.538; *P* = 0.0008; *r* ^2^ = 0.1847), after 2 hours of wear (Q5c-*F*_2,61_: 2.8366; *P* = 0,05; *r* ^2^ = 0.1439), and at the end of the wearing time (Q5d-*F*_4,61_: 11.6696; *P* = 0.00; *r* ^2^ = 0.1999). There is a respective increase of 13% at 2 hours, 8% (mid-day) and 14% (end of the day) in subjects reporting reduced symptoms on V2 compared to V1. With the use of SH drop, 8 out of 10 patients report this symptom as not intense at all. 

### 3.7. Blurry Vision (Q6)

Considering that astigmatism is not in play among study's subjects (exclusion criteria), blurry vision is considered to be related to tear film instability and contact lens dehydration.  Table 7 shows the results. The difference here is highly significant. The use of SH drop helps to reduce almost by half the number of subjects experiencing blurry vision during their contact lens wear. Sixty percent of subjects in this study reported not feeling that symptom at all, after using SH drop before insertion (chi square *F*_4,61_:  13.964; *P* < 0.00). This result shows a strong correlation (*r* ^2^ = 0.2154) and is verified the same way in all care system groups. 

**Figure 1:**
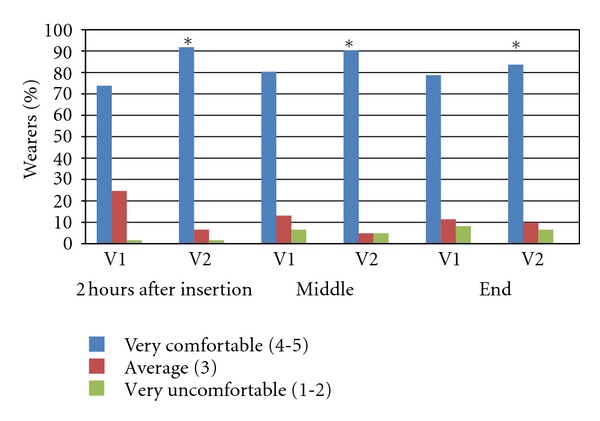
CLDEQ 4, comfort at different moments of the day.

Questions 6 b, c, and d provide more details on this issue by asking how noticeable the blurry vision was at different times: after 2 hours (Q6b), in the middle of the day (Q6c), and at the end of the wearing period (Q6d).  Figure 1 shows the results. There is no statistical difference between subjects at the first two time points (6d). At the end of the day, 15% more patients report no symptom with the use of SH drop (V2) (*F*_4,61_ = 5.4858; *P* = 0.03), even after many hours of wear. Again, this result is the same with all the care system used, with no significant differences among groups. 

### 3.8. Eye Soreness and Irritation (Q7)

At visit 1, 84.7% of the wearers report this symptom as never or infrequently present while 15.3% report it as frequent or constant. At visit 2, only 5.1% of the wearers still experienced this symptom. This is considered a significant difference in favor of the use of the SH drop (chi square *F*_1,61_ = 2.1899; *P* = 0.04; *r* ^2^ = 0.0869). In other words, 10% of the subjects experiencing eye soreness and irritation became asymptomatic with the use of a comfort drop on the lens before insertion. This is a two-thirds reduction (66% less). This difference was not present after 2 hours of wear (Q7b, *P* = 0.90) but significant in the middle of the day (Q7c, *P* = 0.00) and highly significant at the end of the wearing time (Q7d, *P* = 0.00). This was again validated across all groups (A, B, C, D). 

### 3.9. Discomfort Leading to Lens Removal (Q13)

Discomfort felt while wearing contact lenses is one thing. Discomfort that bothers the wearer sufficiently leading to lens removal is another thing. This is the object of Q13 in CLDEQ questionnaire. Results are shown in Table 8. A highly significant difference exists between visit 1 and 2 (chi square *F*_4,61_ = 14.0198, *P* < 0.00), with a strong correlation (*r* ^2^ = 0,2463). At V1, 21% of the subjects admit removing their lenses many times/week because of discomfort, while only 11% report the same at V2. This is a 50% reduction of symptomatic patients with the use of a SH-based drop on the lens before insertion. 

Question 13b considers which cause is most likely to contribute to the discomfort. Ocular discomfort and ocular dryness were, respectively, the number one and two reasons identified by subjects to explain this need to withdraw their lenses. Looking at intensity of these two symptoms, a positive effect of the comfort drop applied before insertion is found. In fact, there is a highly significant difference in the grading of the severity of ocular discomfort and ocular dryness (Q13b, item (a) and (b)) between V1 and V2 (*P* < 0.00 for each symptom). 

### 3.10. Comfort Questionnaire

Table 9 shows the results. There is a significant difference at every time point, but the most obvious difference is during the evening (*F*_4,61_ = 10.943; *P* = 0,00; *r* ^2^ = 0.1817). We find again here the 10% positive difference in favor of the use of SH drops, confirming the CLDEQ results. Over 60% of subjects reported a very comfortable/comfortable lens during the evening, after more than 10 hours of wear, compared with 52% without the use of the drops. This positive effect is already measured in the afternoon with a 7% increase in the number of wearers reporting comfortable instead of average wear. These results indicate that the subjective positive effect from the use of SH drops starts early during the wear and increases over time. 

**Figure 2:**
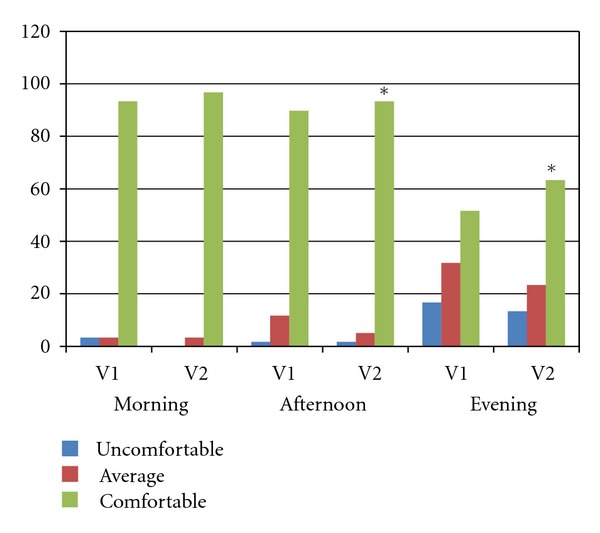
Comfort at different moments of the day (Q2, questionnaire 2).

**Figure 3:**
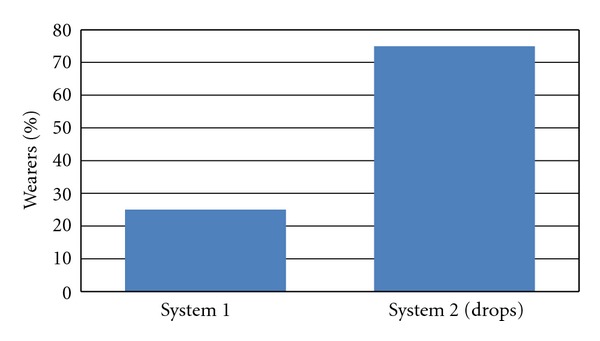
Overall preference at the end of the study.

At the last visit, subjects rated the ease of use of the 2 care regimens they used in the past 2 months (with and without SH drops). Ninety percent (90%) of the subjects rated as very easy or easy both care regimen, without statistically significant differences. The care regimen used at V1 was rated as easy (20%) or very easy (70%), while the second one, including drops before insertion, was rated easy (25%) or very easy (65%). When asked, near 9 out of 10 subjects would recommend the care regimen with SH drops (58.3% strongly and 30% certainly). Only 11.7% of subjects would hesitate (perhaps). None of the subjects would not recommend the system (item 4 and 5). Finally, 75% of subjects would definitely opt for the system with SH drops in the future (see Figures 2 and 3). 

## 4. Discussion

 This study aimed to evaluate the clinical outcome from the instillation of SH drop, as an additional step in the wearing regimen of contact lens wearers, on contact lens related comfort and visual performance over the day. The cross-over design of the study was established to alleviate Hawthorne-like effects as well as sequencing effects. One could argue that the lack of a control group, using another type of lubricating drop, would have been beneficial. Even if this is true, we believe that the cross-over design eliminates any bias that can be raised and helps to determine the real effect from the usage of SH drop instilled in the lens before insertion. 

 The results of the present study prove that sodium-hyaluronate-based drops can be added to the list of products that can help symptomatic contact lens wearers to reduce their overall discomfort (CLDEQ-Q4), but especially at the end of the day (Q4 subquestions), when it matters most. Even if the symptoms remain, symptom severity is greatly reduced with the use of SH drops (CLDEQ-Q5). 

 SH drops also have a positive impact on the subjective visual acuity (CLDEQ-Q6). There is a significant reduction of blurry vision reported by the subjects on visit 2, which means that the use of SH drops probably helps to maintain tear film stability and also contributes to reduce lens dehydration. We have to consider that blurry vision represents the number 2 reason (and the first one for astigmatic patients) to drop out from contact lens wear. 

 In this study, the use of a SH drop initiated a switch for a significant percentage of the wearers from symptomatic to asymptomatic (eye soreness and irritation (CLDEQ-Q7)). For these subjects, discomfort was no longer a reason to remove their contact lenses prematurely (CLDEQ-Q13). More than 60% of wearers reported remaining comfortable in their lenses during the evening, compared with 50% when they did not use the SH drop before insertion (Comfort questionnaire). 

 Clinical findings observed through slit lamp were also improved with the use of SH drops on the lens before insertion. It was observed a reduction in the level of corneal staining, whereas clinically significant hyperemia levels were reduced by more than 50%. Pairing of the results indicates an improvement of TBUT, when subjects are compared to themselves, after the use of the SH drop. These objective findings correlate with subjective reports of increased comfort with the use of SH drops as part of the regular care regimen. They are not surprising considering the nature of the sodium hyaluronate and its known beneficial effects on the ocular surface restoration, namely, on dry eye patients. 

 Consequently, we can say that the use of SH drops before insertion helps to address the two major reasons why patients cease to wear contact lens wear (discomfort and vision) and contributes to a reduction in the physiological impact from contact lens wear. This reproduces the impact of the use of carboxymethylcellulose drops before insertion. This is not anecdotal and should represent a significant step forward to keep patients in the contact lens market. It is easy to realize the impact on wearers, practitioners, and the contact lens industry if, with just one easy additional step before lens insertion, it is possible to keep 10% more of the wearers in contact lenses on an ongoing basis and to improve symptoms of others by 50 to 60%. A single drop put on the lens before insertion can considerably change the contact lens experience for subjects who report ocular dryness. 

 Noncompliance to this new step is certainly not a big issue: instilling one drop before lens insertion was rated easy by a large majority of subjects and 3 out of 4 of them would recommend this to other wearers or their relatives. It is a well-accepted and easy way to improve contact lens wear. 

 Another important aspect of this study is that the positive outcome from the use of SH drops before insertion was verified with multiple types of care systems habitually recommended to wearers. Even subjects using a hydrogen peroxide system found a positive effect from the use of SH drop before lens insertion. Practitioners, therefore, should not hesitate to recommend this step to their symptomatic wearers in order to improve their overall contact lens experience.

## 5. Conclusion

 A sodium hyaluronate drop before insertion of contact lenses improves the overall comfort but its clinical effect is highly significant at the end of the wearing period, during the evening. Its use helps to eliminate or to significantly reduce discomfort symptoms related to contact lens wear and to improve contact lens experience. SH drops also help to maintain more stable vision throughout the day. 

Because of that, it is possible to say that the use of SH-based drops (Blink contacts) before insertion addresses the two major reasons why contact lens wearers drop out from the market. 

 SH drops are compatible with every care system regularly prescribed to wearers. Its clinical effect is the same regardless of the care system used. The use of SH drops before insertion should be considered to improve overall contact lens performance for symptomatic contact lens wearers. 

## Figures and Tables

**Table 1:** Inclusion and exclusion criteria.

Inclusion criteria:	
(i) Age between 18 and 44 years old	
(ii) Successfully wear comfilcon A silicone hydrogels contact lenses on a regular basis (8 hours/day, 5 days/week, x last 3 months)	
(iii) To be myopic (−0.75 to −6.00 diopters) with a refractive astigmatism of less than 1.00 D	
(iv) Present with symptoms of ocular dryness related to contact lens wear	
(v) Be able to provide a legal consent	
(vi) Be available for a period of 2 months	
Exclusion criteria:	
(i) Do not match age criteria	
(ii) Wear of a rigid gas permeable contact lenses	
(iii) Usage of comfort drops more than 4 times/day on a regular basis	
(iv) Part-time contact lens wear (<5 days/week, <8 hours/day)	
(v) Myopia > 6.00D and/or a refractive astigmatism > 0.75D	
(vi) Diagnosis of an active pathological condition or corneal ectasia	
(vii) Chronic use of topical ocular drug(s)	
(viii) Uptake of Omega 3 supplements and/or systemic medications with potential side-effects on ocular dryness	
(ix) Allergy to any product used in this study	
(x) Participation in another clinical study at the same time	
(xi) Unfit to give legal consent	

**Table 2:** Clinical results.

	OD				OS			
	Corneal stg.	Conj. stg.	Hyperemia	TBUT	Corneal stg.	Conj. stg.	Hyperemia	TBUT
	(median)	(median)	(median)	average	(median)	(median)	(median)	Average
V1	0.4 (±0.7)	1.0 (±0.7)	1.5 (±0.7)	5.8 (±3.1)	0.6 (±0.7)	1.0 (±0.7)	1.4 (±0.6)	5.8 (±3.1)
V2	0.0 (±0.7)	0.6 (±0.7)	1.0 (±0.6)	6.3 (±3.2)	0.0 (±0.05)	0.8 (±0.6)	1.0 (±0.6)	6.5 (±3.4)

**Table 3:** Results of the CLDEQ question 4 on discomfort.

	1-2	3	4-5
V1 (no drops) (percent of wearers)	6.7	23.3	70.0
V2 (with drops)	15.0	5.0	80.0

**Table 4:** Intensity of discomfort at different points of the day.

	1-2	3	4-5
V1 (percent of wearers)			
After 2 hours of wear	73.7	24.6	1.7
Middle of the day	80.3	13.1	6.6
End of the day	78.7	11.5	8.2
V2			
After 2 hours of wear	91.8	6.6	1.7
Middle of the day	90.1	4.9	4.9
End of the day	83.7	9.8	6.5

**Table 5:** Occurrence of eye dryness felt during contact lens wear.

	Infrequently	Frequently	Constantly
V1 (percent of wearers)	37.9	32.8	29.3
V2	43.1	48.3	8.6

**Table 6:** Intensity of eye dryness symptoms.

	Overall		After 2 hours of wear		At the end of the day	
Grade/percent of wearers	V1	V2	V1	V2	V1	V2
Not at all intense/not intense	66.7	80.0	91.7	98.3	56.7	71.7
Moderately intense	20.0	11.7	6.7	1.7	16.7	20.0
Intense/very intense	13.3	8.3	1.6	0.0	26.6	8.3

**Table 7:** Blurry vision associated with contact lens wear (Q6).

	Never	Infrequently	Frequently
V1 (percent of wearers)	35.0	40.0	25.0
V2	60.0	28.3	11.7

**Table 8:** Discomfort leading to lens removal.

	Rarely	Once a week	Many times/week
V1 (percent of wearers)	60.0	18.3	21.7
V2	61.7	26.7	11.7

**Table 9:** Subjective comfort at different moments of the day (Questionnaire 2, question 2).

	Very uncomfortable /uncomfortable	Average	Very comfortable /comfortable
V1 (percent of wearers)			
Morning	3.3	3.3	93.3
Afternoon	1.7	11.7	86.6
Evening	16.7	31.7	51.6
V2			
Morning	0	3.3	96.7
Afternoon	1.7	5.0	93.3
Evening	13.3	23.3	63.3
